# Retinoid X Receptors Intersect the Molecular Clockwork in the Regulation of Liver Metabolism

**DOI:** 10.3389/fendo.2017.00024

**Published:** 2017-02-13

**Authors:** Salvatore De Cosmo, Gianluigi Mazzoccoli

**Affiliations:** ^1^Department of Medical Sciences, Division of Internal Medicine and Chronobiology Unit, IRCCS “Ospedale Casa Sollievo della Sofferenza”, San Giovanni Rotondo, Italy

**Keywords:** RXR, circadian, rhythm, clock, liver, metabolism

## Abstract

Liver metabolic pathways are driven by the biological clock, and appropriate timing of 24-h patterns of metabolic gene expression as well as anabolic/catabolic processes with wake-related activity/feeding and sleep-related resting/fasting cycles preserves hepatic healthiness. The interplay among the liver metabolic pathways and the molecular clockwork is geared by the nuclear receptors, and ligand-dependent transcription factors that gauge the cellular nutritional status and redox balance, bind hormones and metabolites, and modulate the transcription of thousands target genes through their DNA-binding domain. Several nuclear receptors in the liver oscillate with circadian rhythmicity, and among these, the retinoid X receptors play a key role in metabolism regulation, intersecting with the cogs of the molecular clockwork.

## Introduction

The regulation of lipid and glucose metabolism in the liver requires cell sensors capable of monitoring the concentration of nutrients and coordinating the enzyme cascades that regulate the synthesis and oxidation of metabolites. In this regard, the nuclear receptors are transcription factors that act as intracellular sensors, which are able to regulate lipid and glucose metabolism. Unlike extracellular receptors, which bind to protein ligands (e.g., growth factors and insulin) at the plasma membrane and activate cytoplasmic cascades, nuclear receptors interact directly with lipophilic ligands and regulate the expression of target genes (1). Members of the nuclear receptor superfamily function as ligand-dependent transcription factors that bind to promoters of specific DNA sequences. The nuclear receptors bind hormones, such as cortisol, melatonin, and 3,5,3′-triiodothyronine, and metabolites, such as lipids, oxysterols, heme, and bile acids, and sense redox balance and nutrient levels in the cell. The nuclear receptors have in common similar domain organizations, especially the DNA-binding domain (DBD) and the ligand-binding domain (LBD) that are crucial in amplifying hormone and metabolite signaling by specifically targeted genes. Binding of specific ligands to the precise receptor prompts ligand-induced conformational variations in the receptor, receptor translocation to the nucleus, receptor dimerization, interaction with target gene promoter elements, release/recruitment of coactivators or corepressors, chromatin remodeling, and finally interplay with the polymerase II complex to start transcription (2).

## The Retinoid X Receptors (RXRs)

The RXR subtypes or isotypes α–γ (NR2B1–3) are encoded by RXRα–γ genes located on chromosomes 9 (band q34.3), 6 (band 21.3), and 1 (band q22–q23), respectively, and are members of the orphan nuclear receptor family since natural ligands were unidentified at the time of their discovery (3). The first candidate natural ligand was 9-*cis*-retinoic acid (9-*cis*-RA), but many researchers were not capable to identify endogenous 9-*cis*-RA in cells either in culture or *in vivo* without existence or addition of its isomer all-trans retinoic acid (ATRA) (3). Other RXR ligands are polyunsaturated fatty acids, such as docosahexaenoic acid (22:6), arachidonic acid (20:4), and oleic acid (18:1), and a saturated metabolite of chlorophyll, phytanic acid. In the nucleus, RXR works as a transcription factor and binds as a homodimer or heterodimer (bound to a different nuclear receptor) to definite 6 bp sequences of DNA in the promoter regions of specific genes (3). The promoter site [response element (RE)] composed of two 6 bp sequences (half-sites) separated by a discrete number of bases to which the RXR–nuclear receptor heterodimer binds [5′-PuG(G/T)TCA-(X)*n*-PuG(G/T)TCA-3′] is determined through binding by the ligand of the nuclear receptor partner. The sequences may be reiterated directly (DR), inverted (IR), everted (ER), palindromic (pal), or disordered in relation to the dimer bound (3).

## Circadian Rhythmicity and Nuclear Receptors

The nuclear receptor superfamily encompasses 49 members, which manage lipid and carbohydrate metabolism and harmonize various features of organ physiology, as well as tissue development and organism reproduction (4). The nuclear receptors are expressed differently in the various tissues (5) and particularly in metabolically active tissues (liver, muscle, and white and brown adipose tissues), some of which show tissue-specific rhythmic fluctuations of expression characterized by 24-h periodicity (circadian) (6). Intermediate metabolism is hallmarked by time-related changes consistent with the daily light/darkness alternation, and periodic circadian variations in the levels of nuclear receptors may prompt rhythmic oscillations of the metabolic pathways (7). The interplay between rhythmic changes of nutrient levels and ligand binding by the nuclear receptors drives periodic variations in downstream transcriptional events steering different metabolism facets. The array of rhythms is driven by the biological clock and in turn the nuclear receptors feedback to the molecular clockwork connecting circadian and metabolic pathways (7).

## The Biological Clock

The organization of about 24-h rhythmicity in organism and cellular physiology is handled in mammals by the circadian timing system, a multilevel hierarchical network comprising central oscillators in the suprachiasmatic nuclei (SCN) of the hypothalamus and self-sustaining oscillators in peripheral tissues. The SCN biological clocks respond to several entraining factors: external, such as the photic inputs perceived by the retinal ganglion cells and conveyed by the retinohypothalamic tracts, and internal, such as temperature, hormones, and metabolites. The SCN neurons drive the peripheral oscillators by way of output pathways represented by hormones (cortisol, melatonin) and autonomic nervous fibers (8). At the molecular level, the biological clock is hardwired by transcription–translation feedback loops (TTFL) revolving one cycle in approximately 24 h and operated by circadian genes and proteins. The positive limb of the loop is worked by the transcription factors Clock and Arntl, which heterodimerize and bind to E-box enhancer elements in the promoters of *Period* (*Per1–3*) and *Cryptochrome* (*Cry1–2*) genes. These genes in turn prompt the negative limb of the loop encoding Per1–3 and Cry1–2 proteins, which interact to form a repression complex that along with casein kinase Iϵ (CKIϵ) translocates back into the nucleus, where it hinders Clock:Arntl transcriptional activity (9, 10). In addition, Clock:Arntl heterodimer induces the circadian expression of *Rev-erb*α and *Rora* genes, which encode the nuclear receptors Rev-Erbα alpha and RORα respectively. In turn, Rev-Erbα negatively controls the rhythmic transcription of *Arntl* gene, competing with RORα at the specific REs (11). The circadian proteins undergo posttranslational modifications, in particular phosphorylation/dephosphorylation and acetylation/deacetylation cycles. Per1–3 and Cry1–2 proteins are substrates for at least three different enzymes operating phosphorylation: CKIδ and CKIϵ, AMPK, and glycogen synthase kinase (GSK) 3β. In particular, phosphorylation by CKIδ/ϵ targets Per1–3/Cry1–2 proteins for degradation and regulates their nuclear translocation, AMPK targets Cry1–2 proteins, and GSK3β targets Arntl (12–15). Acetylation is operated by Clock, which has protein and histone acetyltransferase activity (16), and deacetylation by SIRT1, an NAD^+^-dependent protein, and histone deacetylase assuring high-magnitude circadian transcription of several core clock genes, including *Arntl, Per2*, and *Cry1*. SIRT1 offsets the acetyltransferase activity of Clock, binds Clock–Arntl heterodimer in a circadian manner and promotes the deacetylation and degradation of Per2 (17–19).

## RXRs and the Nuclear Receptor Superfamily

The molecular clockwork controls the expression of numerous clock-controlled genes and tissue-specific output genes, so that about 15–20% of the transcriptome oscillates with circadian rhythmicity and manage the rhythmic changes of cell processes (metabolism, redox and oxphos balance, autophagy, cell cycle, DNA damage response) and tissue functions (liver and renal physiology, heart function, endocrine gland secretion) (20).

Among the clock-controlled genes, some encode nuclear receptors oscillating with circadian pattern in metabolically active tissues and specifically in the liver, such as RXRs, constitutive androstane receptor (CAR), estrogen-related receptor α, β, and γ, farnesoid receptor (FXR) α and β, glucocorticoid receptor, Nur-related protein 1 (NURR1), peroxisome proliferator-activated receptor (PPAR) α, δ/β, and γ, retinoic acid receptor (RAR) α, β, and γ, small heterodimeric partner, and thyroid hormone receptor (TR) α (6). Among these, a particularly intriguing role is played by RXRs, forming heterodimers with numerous members of the nuclear receptor superfamily including PPARs, RARs, FXRs, LXRs, TRs, CAR, NURR1, and vitamin D3 receptors (VDRs) (3, 21–23). Indeed, RXRs unrestrainedly heterodimerize with these members of the nuclear receptor superfamily, and by means of these interaction, RXR ligands, also called rexinoids, either transcriptionally stimulate *per se* the permissive subclass of heterodimers (PPAR/RXR, LXR/RXR, FXR/RXR, and CAR/RXR) or synergize with partner ligands in the non-permissive subclass of heterodimers (RAR/RXR, VDR/RXR, and TR/RXR) (24–26). The permissive heterodimers become transcriptionally active in the presence of either an RXR-selective ligand (rexinoid) or a nuclear receptor partner ligand, and the simultaneous presence of both RXR and partner receptor ligands results in a cooperative, synergistic response (1). By contrast, non-permissive heterodimers are unresponsive to rexinoids alone, but these agonists superactivate transcription by synergizing with partner agonists (27).

The TR/RXR heterodimer is generally defined as non-permissive, so that RXR is supposed to act as a silent partner not capable to bind specific ligands and full activity of the TR/RXR heterodimer is achieved only upon 3,3′,5-triiodo-l-thyronine (T3) binding and not by the RXR agonist. Anyway, studies performed with a sensitive derepression assay system showed that the RXR component can bind its ligand *in vivo* in a TR/RXR heterodimer without straight activation of the TR/RXR heterodimer, but with the TR dissociating from inhibitor(s)/corepressor(s), as a minimum in a temporary or dynamic manner, leading to TR-mediated repression in the absence of a ligand and/or TR-mediated activation upon ligand binding (28). Besides, 9-*cis*-RA was proved to induce conformational changes within the TR/RXR complex and to act as an allosteric repressor of transactivation (29).

Retinoid X receptor heterodimers with PPAR, RAR, VDR, and TR consist of two directly repeated (DR) half-sites separated by one, two or five, three, and four bases (*n*), respectively, typically with RXR in the 5′-position. When the RXR heterodimer with RAR is bound to a DR-1 RE, RXR can lodge either to the 5′- or 3′-position. The RXR homodimer favorably binds two 5′-(A/G)GGTCA-3′ half-sites disjointed by one base (DR-1).

Kinases modulate the function of RXRs homo- and heterodimers. In particular, AMPK activators hinder FXRRE-bound RXR/FXR heterodimers exclusively acting on FXR hindering FXR coactivator recruitment to promoters of FXR-regulated genes and FXR transcriptional activity, whereas RXRs are not impacted (30). On the other hand, studies performed in cancer cells showed that RXRs are targeted by the CK1 family, and precisely RXR is bound and phosphorylated by CK1α in an agonist-dependent manner (31), as well as by GSK3β, with modulation of cell predisposition to RXR agonist-induced growth arrest and apoptosis and support of cell survival (32).

## RXRs and the Biological Clock

**Figure 1:**
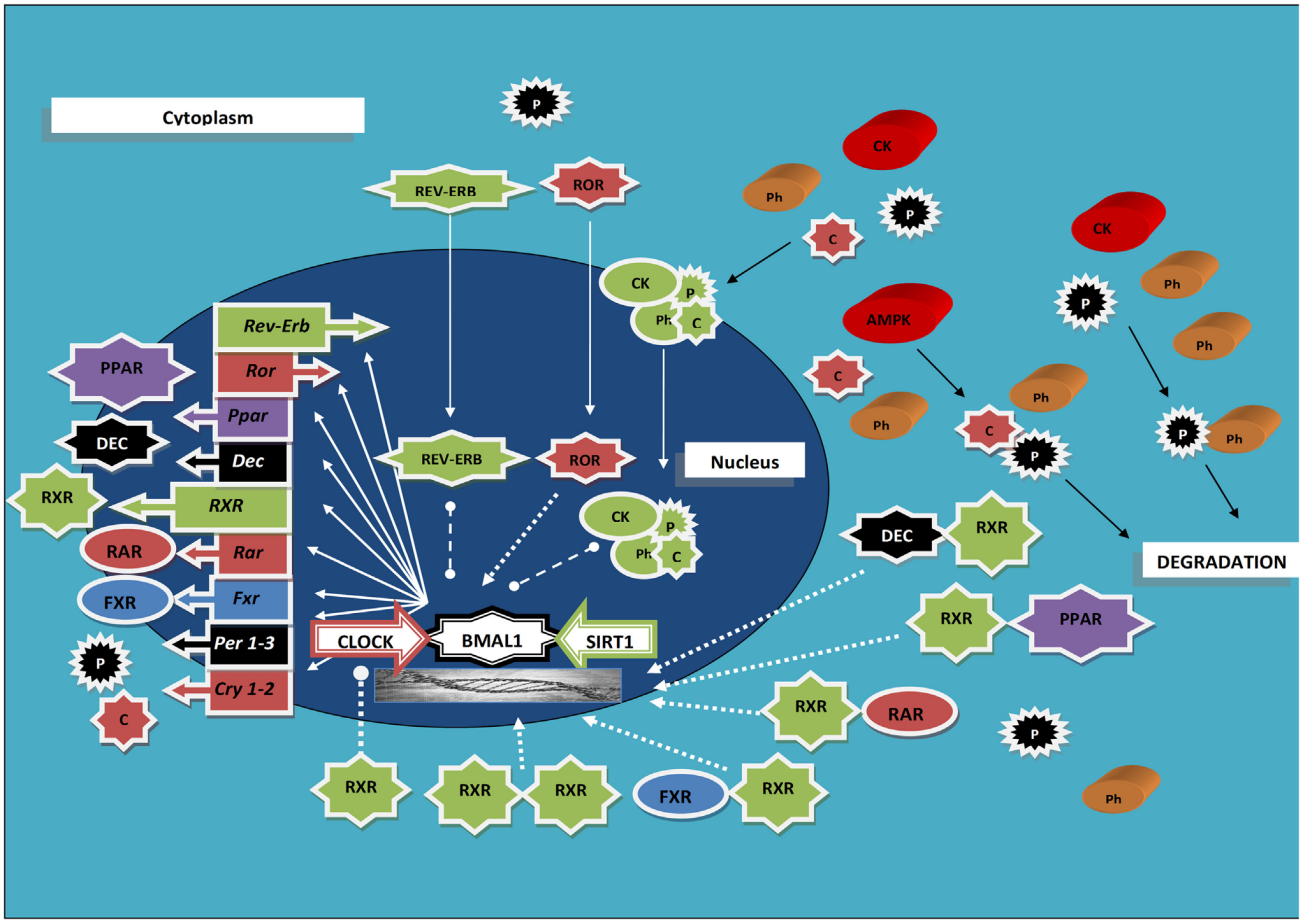
**Scheme rendering the interaction between the molecular clockwork, the retinoid X receptors, and other nuclear receptors oscillating with circadian rhythmicity; see text for acronyms explanation**. Ball-ended dashed lines indicate inhibition; arrow-ended dashed lines indicate activation; arrow-ended continuous lines indicate translocation; black lines indicate modification.

A tight interplay has been evidenced between RXRs and the cogs of the molecular clockwork. RXRs are capable to join with members of the positive limb of TTFL with an interaction influenced by circulating factors, and RA was found able to hinder clock genes transcription both in cultured muscle cells and in cardiovascular organs from intact animals (33) (Figure 1). Precisely, RXRα interacted in a ligand-dependent manner with the basic helix-loop-helix proteins Npas2 and Clock (the interaction strength boosted up to 15-fold upon specific ligand binding), but not with Bmal1, hindering, as a minimum *in vitro*, transcriptional activity of Clock/Npas2–Bmal1 heterodimers at the promoters of clock genes, and RA injection prompted small phase shifts of peripheral clocks in the heart and aorta (33). Besides, ATRA, 9-*cis*-RA, and 13-*cis*-RA were found capable to entrain circadian rhythmicity in cultured fibroblasts expressing Per2-luciferase (34), and retinoic acid signaling elicited central clock resetting upon light sensing (35, 36). Moreover, RXRα–cofactor proteins interaction as well as RXR nuclear receptors heterodimerization and ligand-dependent transactivation by RXR was found to be modified and inhibited, respectively, by the basic helix-loop-helix proteins differentiated embryo chondrocyte (Dec) 1 and Dec2 (37). The expression of Dec1–2 is driven by the biological clock, oscillates with circadian pattern in the mouse SCN and liver peaking in the subjective day, and takes part in the molecular clockwork, thereby blocking Clock/Arntl-induced transactivation of the mouse *Per1* promoter through direct protein–protein interactions with Arntl and/or competition for E-box elements (38, 39). Interestingly, experiments performed in the rat showed that insulin prompts the transcription of the *Dec1* gene *via* a phosphoinositide 3-kinase pathway, further supporting a direct link between intermediary metabolism and the molecular clockwork (40).

Besides, the interplay between RXRs and the biological clock showed up remarkably in an animal model of liver regeneration, represented by partial hepatectomy in RXRα-null compared to wild-type mouse. Hepatocyte proliferation showed approximately 20-h delay after partial hepatectomy in liver RXRα-deficient mice and numerous pathways were dwindled, in particular circadian cell cycle regulation. Furthermore, in accordance with the evidence that PPARα regulates the transcription of both Arntl and Rev-erbα *via* PPAR-response and *cis*-regulatory elements in their promoters (41), the PPARα/Arntl/Rev-erbα/P21 pathway was disrupted, and Cry1/Cry2 and Wee1/Per1 expression as well as the expression and regulation of cyclin D1/cyclin-dependent kinase (Cdk) 4, cyclin E1/Cdk2, cyclin A2/Cdk2, and cyclin B1/Cdk1 were deranged in regenerating RXRα-null livers (42).

Accordingly, studies performed on the hippocampus specimens of Holtzman rats harvested at 4-h intervals for 24 h showed clock-responsive canonical and non-canonical E-box elements and RORE sites in the regulatory regions of genes encoding RARs and RXRβ, as well as retinoic acid response elements (RAREs) and RXREs in *Bmal1* and *Per1* promoters (43).

Furthermore, an interplay between the PPARs/RXRα-regulated system and the molecular clockwork was shown. PPARα/RXRα modulated Period and Cryptochrome genes transcription driven by Clock/Bmal1 heterodimers (44). In turn, Clock/Bmal1 heterodimers binding to consensus E-box elements modulated PPARs/RXRα-dependent transcription through peroxisome proliferator response element of target genes, comprising those encoding acyl-CoA oxidase (AOX), lipoprotein lipase, acyl-CoA synthase, cellular retinol-binding protein II, and 3-hydroxy-3-methylglutaryl coenzyme A (HMG-CoA) synthase (44).

## RXRs and Transcriptional Activity

Retinoid X receptors as homodimers or heterodimerizing with RARs manage the signaling pathways and genetic transcriptional programs dependent on retinoids, crucial for vertebrates development and homeostasis (1, 3, 45). Non-steroid nuclear receptors bind to a core recognition element represented by the consensus sequence 5-AGGTCA-3, but RXRs and RARs minimally bind to this consensus site as monomers (45). Heterodimerization boosts their cooperative affinity and selectivity for RAREs, which are set up as coupled binding sites, so that RXR/RAR heterodimer binding to RAREs is more proficient respect to the homodimeric forms of RXR or RAR (1, 45). The nuclear receptor superfamily DBD maintains a highly conserved core sequence comprised of two zinc-nucleated modules and two α-helices folding into a single globular domain, with approximately 40% amino acid identity over a 67-residue region. In spite of the use of a highly conserved receptor DBD and a single major core binding sequence, complex DNA sites can be exploited with better selectivity by means of RXR forming combinatorial receptor heterodimers so that eventually two different ligands can co-regulate transcription from the same gene-regulatory element (45). The nuclear receptors DBDs can produce the same pattern of DNA selectivity and dimerization as the full-length receptors (3, 45), whereas the LBDs dimerization function of some receptors can additionally stabilize some dimeric assemblies but has no selective role for RE recognition (3, 45). The nuclear receptor superfamily members exploit the same consensus AGGTCA sequence, and transcriptional control selectivity is furthermore warranted by multiple arrangements of AGGTCA sites in the REs. RXR/RAR heterodimers preferentially bind to two AGGTCA sites set up in a direct repeat (DR) configuration hallmarked by inter-half-site spacings of 1–5 bp acknowledged as DR1–DR5, respectively (3, 45). The spacer size differentiates more respect to the sequence, with the maximum affinity binding sites for each RXR heterodimer and the discriminating binding of DRs based on inter-half-site spacings rated on a scale of 1–5. Other members of the nuclear superfamily link powerfully to inverted and everted repeats of core hexamer so that the usage of direct repeat geometry is less crucial (3, 45).

## RXR Heterodimers

Combinatorial nuclear receptors are characterized by an amazing recognition mechanism that permits them to decipher a binding site geometry in addition to its sequence, with polarity adopted by the subunits of the bound receptor heterodimer representing an associated feature of any asymmetric RE. The RXR/RAR complex can assume diverse polarities when bound to its different REs, with different functions on the heterodimer dependent on ligand and corepressor binding. In this context, although the two hexameric binding sites may have the same sequence, the non-symmetric RXR/RAR complexes permits the position of every receptor to be distinguished as upstream or downstream (3, 45). A preceding structural analysis of the RXR/TR DBD complex linked to a DR4 thyroid RE permitted the direct configuration of the polarity related to that complex. The asymmetric assembly was recognized through the collaboration between DBD subunits, which arises exclusively with a 4-bp inter-half-site spacing length and RXR positioning upstream of TR (3, 45). The RXR/RAR heterodimer, whose RE repertoire is relatively small, mediates transcriptional control with low selectivity through DR1, DR2, and DR5 sequences located in REREs, whereas the RXR/TR complex is only restricted to DR4. A great flexibility measure of their DBDs in forming valid interaction surfaces with different effects on target gene regulation is hinted by the multiple high-affinity DNA-binding targets (3, 45). *In vivo*, RXR/RAR heterodimers on DR1 inhibit transcription with or without occurrence of specific ligands. The complex structure allows the DR1 to identify the polar assembly of the RXR and RAR DBDs as well as the aptitude of a DNA regulatory element to work as a typical allosteric ligand, triggering new conformations and/or interactions eventually augmenting its own binding.

## RXRs and DNA REs

DNA REs induce two different types of allosteric changes on nuclear receptors: (i) conformational changes ensuing within a DBD, such as the T-box α-helix deformation, prompting increased RXR binding DNA; (ii) DNA structures reshaping to facilitate protein–protein contacts. Effective RXR:RAR interactions induce a considerable improvement in their DNA affinity. The REs effectively impinge on the interactions of full-length nuclear receptors with their ligands, corepressors, coactivators, AP-1, and other players that can impact gene expression (3, 45). Other factors are represented by protein flexibility and DNA surfaces exactly changing on a DNA site. The retinoid REs inter-halfsite spacing identifies a fixed geometry for a pair of nuclear receptors to intermingle on, with one nucleotide change in the spacer inducing RXR and its partners to rotate ~35° around the double helix and be dislocated from each other by 3.4 Å. Consequently, RXR DBD must retain several fixed and different surfaces to put up its many combinatorial interactions, or utilize a small number of adaptable protein elements that can adjust to the rotations, displacements, and polarity of these binding sites with one or more receptor partners. Besides, the numerous RXR combinatorial complexes must be prearranged only when needed at particular control sites, to avoid wasting, considering the numerous pairwise interactions that RXR can form with its partners and the crucial role played by the DNA in the definition of the protein structures entailed for dimerization (3, 45).

## RXRs’ Multimerization

In the absence of specific ligands RXRs expressly assemble as tetramers, which disengage when a ligand is bound, causing dimerization surface changes and development of RXR homodimers or heterodimers if adequate partners are present. In particular, ligand binding triggers conformational changes that modify receptor multimerization capability and changes RXRs’ homo-/heterodimerization as well as cofactor-binding surface (mostly related to helices 10/11 and 12 reshuffle) inducing a ligand prompted shift to act as an activator and not as a repressor of gene transcription (46). Other than as receptor dimers, RXRs can also bind to DNA as larger protein complexes (trimers, tetramers, and pentamers) assembling supportively on REs containing appropriately repeated half-sites and crucially guaranteeing unambiguous DNA recognition. In particular, RXR tetramers display important functional plasticity and gather on REs hallmarked by varied half-site alignments and arrangements. The capability of RXRs to create tetramers and correlated oligomers seems to add to the synergistic transcriptional activation observed when multiple, spatially separated REs locate into a single promoter. RXRs oligomer formation contributes to isoform-specific promoter utilization and allows recognition of DNA sequences not recognized by receptor dimers (47).

## RXRs and the Biological Clock in Liver Metabolism

The hepatic metabolic pathways managing glycolysis/gluconeogenesis, lipogenesis/fatty acid oxidation, and protein biosynthesis are essential for glucose, lipid, and amino acid homeostasis. These divergent metabolic processes require temporal separation warranted by the biological clock, which drives rhythmic fluctuations of liver transcripts encoding rate-limiting enzymes and regulators of major metabolic processes, so that metabolic diseases ensue when the ordered sequence of activation of the numerous metabolic pathways is deranged (48, 49). A huge number of hepatic metabolites changes with 24-h periodicity and, for example, the circadian regulation of hepatic function sets up the alternation of glycolysis and gluconeogenesis, with the highest levels of the former in the morning and the lowest levels of the latter in the evening in nocturnal respect to diurnal species. Among the factors which RXRs are capable to interact with, Clock plays a key role in the molecular clockwork and liver metabolism as well, as evidenced by metabolic phenotyping studies performed on mice bearing a dominant negative mutation in the Clock gene (ClockΔ19 mice), which show hyperlipidemia, hyperglycemia, and hepatic steatosis (50). Other important RXRs heterodimerization partners involved in liver metabolism are the PPAR family members, which show 24-h oscillations in mouse liver (6). PPARα, the prevailing hepatic isoform, stimulates fatty acid catabolism by peroxisomal β-oxidation and mitochrondrial β- and O-oxidation in liver (3) and peaks in the early evening, oscillating in phase with the PGC-1α (51) and lipin 1, which magnifies the PPARα/PGC-1α module effects, upholding fatty acids utilization in early nocturnal feeding phase of rodents and anticipating hepatocyte capability to increase energy support for physical activity needs (52). Besides, PPARγ plays an important role in regulating glucose metabolism and fatty acid homeostasis: its functional activity depends on the establishment of RXR/PPARγ heterodimers, which can be activated not only by PPARγ agonists, such as anti-hyperglycemic and anti-diabetic thiazolidinediones, but also by RXR modulators, in particular partial agonists and even antagonists more than full agonists, which cause severe side effects (53).

Other important RXR partners are two nuclear receptors managing the transcription of genes enriching signaling pathways that regulate intestinal and hepatic lipid homeostasis: the LXRs, which control whole-body cholesterol, fatty acid, and glucose homeostasis, and the FXRs, which bind bile acids and are entailed in feedback inhibition of bile acid synthesis and cholesterol catabolism (54). RXR/LXR heterodimers manage cholesterol homeostasis controlling cholesterol transport and catabolism and triacyl glycerol synthesis gene regulation triggering ATP-binding cassette transporters (such as ABCA1) for cholesterol and phospholipids efflux from intracellular receptor stores to extracellular acceptors, sterol responsive element binding protein 1c, a transcription factor controlling fatty acid synthesis, apolipoproteins ApoD and ApoE, lipoprotein-modifying enzymes such as cholesterol ester (CETP), and phospholipid transfer proteins (3). Rexinoid agonists binding to RXR/FXR heterodimers act as antagonists to reduce DNA binding and coactivator recruitment (3). In its side, NURR1 prompts fatty acid-binding protein 5 *via* its promoter NBRE independent of RXR, whereas CAR is involved in response to endobiotic or xenobiotic challenge with induction of P450 enzymes (3).

## RXRs and Therapeutic Potential

The reciprocal modulation deriving from the interplay between the ticking of biological clocks and the circadian fluctuations of nutrients and metabolites in the extracellular as well as intracellular milieu highlighted at present by animal experiments suggests a critical role played by nutrient sensors and RXRs as well as other rhythmically oscillating nuclear receptors in body physiology as well as in pathophysiological mechanisms of disease (55). In particular, RXR agonists (e.g., bexarotene) impact target genes of numerous permissive partners hinting that these molecules may pharmacologically influence numerous metabolically important pathways *in vivo* (56). Accordingly, RXR liver-specific deletion in mice provokes changes in all metabolic pathways regulated by RXR heterodimers, suggesting RXRs are essential and pleiotropic actors (57). Anyway, even if RXR agonists’ multipotentiality hints therapeutic significance on several important signaling pathways and provides boosted effectiveness by permissive heterodimers panactivation, poor selectivity represents an important drawback. Furthermore, considering that RXR agonists trigger hypertriglyceridemia and hypothyroidism in animals and humans, more appropriate therapeutically valuable targets could be represented by RXRs’ heterodimeric partners (58).

## Conclusion

Various ligands ranging from cholesterol to fatty acids and fat-soluble vitamins bind to RXR heterodimers and the cell and context-dependent interaction of the ligand–receptor complexes with co-regulators induces modulation of gene networks, which impact transcriptional and posttranscriptional regulatory factors managing intermediary metabolism and involved in metabolic disorders pathophysiology when deranged. RXR modulators function as agonists, partial agonists, and inverse agonists or antagonists depending on the structure of the ligand–receptor complex, and heterodimer-selective rexinoids could represent future valuable therapeutic tools for metabolic diseases. Accordingly, RXR heterodimers and their interplay with the biological clock are crucial in the time-related regulation of metabolic pathways and represent future druggable targets.

## Author Contributions

All the authors listed have made substantial, direct, and intellectual contribution to the work and approved it for publication.

## Conflict of Interest Statement

The authors declare that there are no conflicts of interest with respect to the authorship and/or publication of this article.
